# Simultaneous Training for Children with Autism Spectrum Disorder and Their Parents with a Focus on Social Skills Enhancement

**DOI:** 10.3390/ijerph13060590

**Published:** 2016-06-14

**Authors:** Hiroko Okuno, Tomoka Yamamoto, Aika Tatsumi, Ikuko Mohri, Masako Taniike

**Affiliations:** 1 United Graduate School of Child Development, Osaka University, Osaka 565-0871, Japan; ikuko@kokoro.med.osaka-u.ac.jp (I.M.); masako@kokoro.med.osaka-u.ac.jp (M.T.); 2 Molecular Research Center for Children’s Mental Development, United Graduate School of Child Development, Osaka University, Osaka 565-0871, Japan; t-yamamoto@kokoro.med.osaka-u.ac.jp (T.Y.); aika-tatsumi@kokoro.med.osaka-u.ac.jp (A.T.)

**Keywords:** autism spectrum disorder (ASD), social skills, family functioning, parental confidence

## Abstract

The objective of this study was to evaluate the effectiveness of simultaneous training for children with autism spectrum disorder (ASD) and their parents, with a focus on social skills enhancement (STSSE) by evaluating behavioral changes in children with ASD and changes in family functioning. STSSE was conducted on 17 children of elementary school age with ASD and their parents. Changes in scores on the social skills scale for education (SS-scale), the child behavior checklist, the Feetham Family Functioning Survey (FFFS), and the confidence degree questionnaire for families (CDQ) were used to assess the effectiveness of STSSE. Improvements were seen for “Communication Skills” on the children’s SS-scale (*p* = 0.029). Significant improvements were seen in the mothers’ FFFS scores for “The 4th factor: illness and worries” (*p* = 0.016) and in the median CDQ scores for one of 18 items after STSSE (*p* = 0.01). Although additional studies with larger sample sizes will be necessary before these findings are generalizable, the positive changes seen in both parents and children as a result of STSSE are promising.

## 1. Introduction

According to the American Psychiatric Association (2013) [1], autism spectrum disorder (ASD) is characterized by deficits in social communication and the presence of restricted, repetitive, and stereotyped interests and behaviors. Deficiencies in social skills can impede a child’s ability to establish and maintain satisfying peer relationships, which in turn is associated with low self-esteem [2]. Children with ASD display markedly different clinical presentations with regard to social and communication impairments [3], and those with more impaired social skills tend to be associated with a high prevalence of school maladjustment, dropout, and adolescent health problems, such as **s**ocial attributions and depression [4,5]. In addition, the families of children with ASD with such social impairments are often perplexed about their child's characteristics.

In Japan, only a few reports have discussed parent training (PT). One of the most effective PT programs was developed more than 20 years ago at the University of California, Los Angeles (UCLA). This PT program and has been used in numerous countries for changing parenting behaviors among parents of children with attention deficit hyperactivity disorder (ADHD) [6]. In order to instruct parents on how to deal with their ADHD children effectively, the UCLA PT program contains essential information such as characteristics of the disorder and treatment strategies. PT that focuses on reducing family conflict through alternative intervention strategies for parents has also been developed [7]. We recently reported that PT with smaller groups and shorter schedules (PTSS) could be useful for reducing problematic behaviors in children with ASD and ADHD and for increasing the confidence of mothers providing care [8].

However, PT does have some limitations. For example, it has not been shown to have a significant effect on behaviors of children with ASD, particularly in terms of externalizing problems. Therefore, a mainstream treatment approach in which children undergo direct training in social skills, problem solving, and anger management has been developed [9,10,11,12]. Some reports have proposed applying social skills training (SST) programs for both children with ASD and their families [13,14,15,16], while DeRosier *et al.* [17] reported a program in which parents who helped affected children with their homework participated together with their child only during the introduction of their session. In another program reported by both Ventola *et al.* [18] and Solomon *et al.* [19], parents attended a semi-structured, concurrent, psychoeducational training meeting during the child’s sessions. Although it is important for parents to gain the social skills and knowledge necessary to promote their child’s social development, to the best of our knowledge, no training programs in which parents learn these skills in a systemic manner have been reported.

Therefore, in the present study, simultaneous training for children with ASD and their parents with a focus on social skills enhancement, which combines SST for high-functioning children with ASD of elementary school age and their parents, was applied in small groups of four to five members. STSSE, the primary focus of which is helping children with ASD acquire social skills, is unique in that it is both an SST program for children and a PT program for parents. STSSE uses a variety of behavioral principles to help enhance the social functioning of children with ASD. An STSSE course, which is simultaneously carried out for children with ASD and their parents, comprises 10 consecutive sessions and can be completed within six months. Parental sessions focus on helping parents gain an understanding of their child’s social skills, including the areas in which they may be lacking, and on assisting their child in adapting to both the home and school environment.

In a previous study, maternal well-being was measured using both adverse maternal outcomes—such as depression and parenting stress—and favorable maternal outcomes—such as life satisfaction and overall psychological well-being [20]; however, assessing only maternal well-being is insufficient for the assessment of family functioning. Enforcement of self-care behavior in families caring for children with ASD and assessment of family functioning as a unit, are also necessary. In particular, a family systems perspective [21] that includes fathers, mothers, and other family members of children with ASD should be utilized. Such a perspective should ask questions such as “How do family members perceive support given and received?” and “How does congruence among family members or the lack thereof contribute to the well-being of each member?” [20]. To our knowledge, only a few studies have evaluated the effects of PT on family functioning among both parents and other family members of children with ASD.

Therefore, the purpose of this study was to evaluate the effectiveness of STSSE for children with ASD and their parents by evaluating behavioral changes in children with ASD and changes in family functioning, including both parents and other family members.

## 2. Materials and Methods

### 2.1. Participants

Explanatory leaflets regarding STSSE were distributed to the families of children with ASD at the outpatient clinic of Osaka University Hospital; 17 children and their mothers were subject to the study. All children were diagnosed with ASD in the outpatient clinic according to Diagnostic and Statistical Manual of Mental Disorders (5th edition) guidelines. All children were aged from seven years eleven months to ten years ten months, and nine had comorbid ADHD. Informed consent was obtained from all children and parents before the study began.

To evaluate the effectiveness of STSSE, parents were instructed not to start any new or change any ongoing medications or interventions for their children during the program. Parents who had previous PT experience or showed serious psychological conditions, including a history of child abuse, or children with cognitive deficit (intelligence quotient/developmental quotient (IQ/DQ) lower than 60) were carefully excluded by the research team, which consisted of the attending doctors, a nurse, and psychologists. The attendance rate among the 17 children and their parents in the STSSE sessions was 90%. When these children and parents could not be present in some sessions, they were supplemented with the content and material of the missed sessions individually.

The participants’ demographic data are shown in Table 1. Mothers (mean age ± standard deviation (SD), 40.7 ± 3.3 years) were the primary caregivers in all cases, and all lived with a partner. Of the 17 mothers, six participated in STSSE sessions together with the children’s fathers. Eight of the 17 children had siblings, some of whom also experienced trouble at school or quarreled with their siblings.

### 2.2. Characteristics of Children

The clinical characteristics of the children who participated in the study are shown in Table 2. Developmental levels were estimated using one or more of the following developmental test batteries: to assess the intellectual quotient (IQ), the Wechsler Intelligence Scale for Children, Third Edition or the Tanaka–Binet Intelligence Scale V [22]; and to assess the developmental quotient (DQ), the Kyoto Scale of Psychological Development [23], a scale widely used in Japan. The resultant IQ/DQ of the children ranged from 60 to 101. The main problematic behaviors were related to social and communication impairments in addition to hyperactivity/impulsion, difficulty in following directions, and restlessness/attention deficit. We then synthetically assessed each participant’s degree of comprehension regarding the content of STSSE, IQ/DQ, autism grade, *etc.*, in order to select appropriate candidates.

### 2.3. STSSE Procedure

STSSE was conducted for small groups of 4 to 5 participants by a registered nurse and clinical psychologists trained in the original SST procedures. Each group member was selected in order of application. A single STSSE course comprised 10 consecutive sessions and was completed within six months. Each session lasted 90 min and was held every 1 to 2 weeks. To maintain the quality of STSSE, two researchers assisted the leader, monitored the contents of each session, and recorded the parents’ remarks and reactions. The records of each session were analyzed and discussed by the researchers, child neurologists, clinical psychologists, and a registered nurse.

### 2.4. STSSE Content

The STSSE content used in this study was formulated based primarily on the original report of SST programs that described “recognition and understanding of emotion; theory of mind; and executive functions/real life-type problem solving” [19]. The children’s program included teaching, modeling, role-playing, rehearsal, feedback, and homework related to the target skills, which were taught to the children by instruction and modeling. Games designed to use the target skills were then played among the children following the role-play with their trainer.

In reference to the programs developed for parents of children with ASD by Butter *et al.* [24], some new items were added, including training for parenting on communication skills, understanding the children’s inappropriate social skills, and helping the children adapt to their home and school environments. The sessions for parents comprised group discussion, role-play, watching a videotaped recording of their sessions, and weekly homework assignments [25,26].

The content and points of intervention for the 10 sessions comprising a course are shown in Table 3.

### 2.5. Evaluation Methods

The effectiveness of STSSE was assessed using the following four scales: social skills scale for education (SS-scale) [27], child behavior checklist (CBCL) [28], the Japanese version of the Feetham Family Functioning Survey (FFFS) [29], originally developed by Feetham *et al.* [30], and the confidence degree questionnaire (CDQ) for families. All mothers and their partners were asked to complete these three scales (CBCL, FFFS, CDQ) separately on two different occasions; the first within one month prior to starting STSSE, and the second within one month after finishing the last STSSE session. The SS-scale was evaluated by the teacher who was in charge of the target child. In addition, the mothers’ comments at the end of each session were recorded and analyzed.

#### 2.5.1. SS-Scale for Education

The SS-scale scoring system consists of the following four factors, each scored on a four-point Likert scale: “Group Behavior Skills”; “Self-Control Skills”; “Friends and Relationships Skills”; and “Communication Skills” [27]. Since this scale has not been standardized, only changes in each sub-item score were used for evaluation (mean score ± SD: 10.1 ± 3.0). When the SS-scale score is increased after PT compared with before PT, the child’s social skills are considered as being improved.

#### 2.5.2. CBCL Japanese Version

The CBCL is a scale designed to evaluate child behavioral problems [28]. Responses are scored on a 3-point Likert scale as follows: 0 = Not true; 1 = Somewhat or sometimes true; and 2 = Very true or often true. The school-age checklist is composed of 120 questions.

#### 2.5.3. FFFS, Japanese First Version

In the FFFS, family functioning is defined as activities and relationships, including growth, safety, socialization, economic stability, and education, among and between individuals, the family, and the environment that enable a family to maintain itself and meet the needs of family members. The FFFS is designed to evaluate family functioning [29,30] by assessing relationships between the family unit and the individual, the family subsystems, and the broader social unit. The FFFS is composed of 25 items, each scored on a seven-point Likert scale. Each item includes the following two questions: (a) ”How much family functioning do you currently have?”; and (b) “How much family functioning would you like to have?” The absolute value of the difference between the “*a*-score” and the “*b*-score” is calculated as the “*d*-score”. The *d*-score represents the discrepancy between perceived and desired family functioning, with a lower *d*-score indicating better family functioning.

#### 2.5.4. The Confidence Degree Questionnaire (CDQ) for Families

This scoring system comprises 18 items (Table 4) that were modified from the original report by Iwasaka *et al.* [25] to incorporate the parents of children with ASD. Each CDQ item is answered on a five-point Likert scale. Since this scale has not been standardized, only changes in sub-item scores were used for evaluation. When the CDQ score is increased after compared with before STSSE, the parent’s confidence in relating to their children is considered to be increased. Replies to the questionnaires were not requested from nine fathers who participated in the first year of study; therefore, only the CDQ scores of mothers were subject to analysis.

#### 2.5.5. The Mothers’ Comments

The mothers’ comments at the end of each session were recorded and analyzed.

### 2.6. Ethical Considerations

All subjects gave their informed consent for inclusion before they participated in the study. The study was conducted in accordance with the Declaration of Helsinki, and the protocol was approved by the Ethical Review Board of Osaka University Hospital (project identification code: 10007-5).

### 2.7. Statistical Analysis

Data were analyzed using SPSS 20.0 for Windows (SPSS Inc., Tokyo, Japan). All scores were compared using the Wilcoxon signed-rank test. *p <* 0.05 was considered to indicate statistical significance.

## 3. Results

### 3.1. Changes in SS-Scale for Education

**Figure 1:**
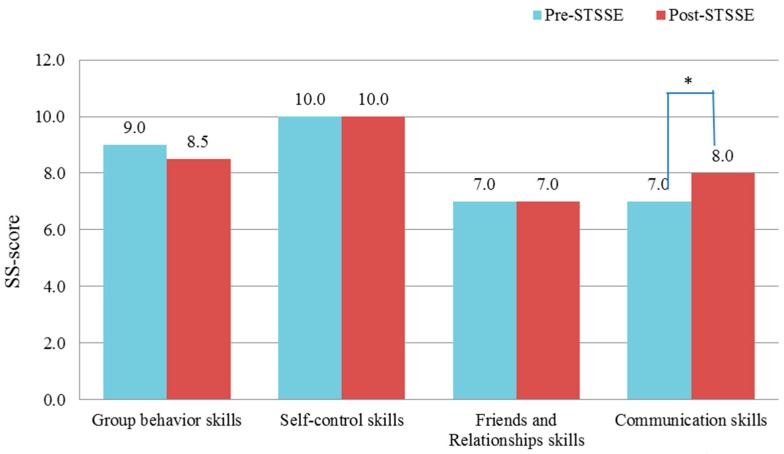
Changes in median social skills scores for children before and after simultaneous training with a focus on social skills enhancement (STSSE).SS-scale: social skills scale for education. *p* < 0.05.

Increases were seen in all SS-scale scores for education after STSSE. In particular, significant increases were seen in the scores for “Communication Skills” (*p =* 0.029; Figure 1), which includes the sub-items “Hearing”, “Speaking”, “Assertion”, and “Talking”. These findings suggest that STSSE is particularly helpful in these aspects of communication.

### 3.2. Changes in CBCL Scores

**Figure 2:**
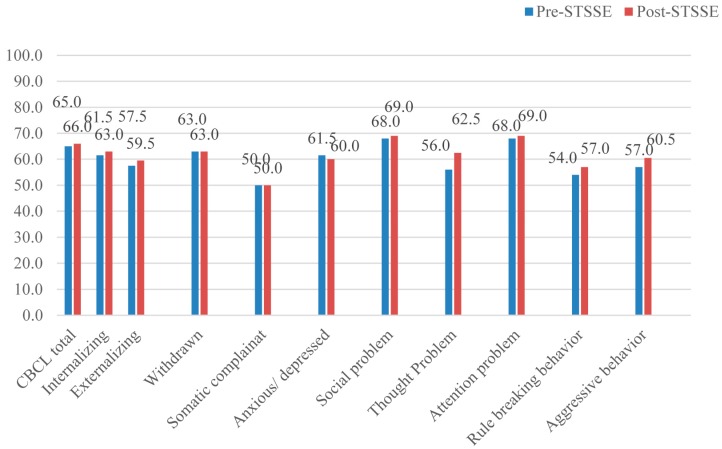
Changes in median child behavior checklist (CBCL) and Externalizing and Internalizing *T*-scores before and after STSSE.

The CBCL total *T*-score and the Externalizing and Internalizing *T*-scores were unchanged after STSSE (total *T*-score: 65.0 to 66.0; Externalizing *T*-score: 57.5 to 59.5; Internalizing *T*-score: 61.5 to 63.0) (Figure 2). No significant differences were evident among any of the children.

### 3.3. Changes in FFFS Scores

**Figure 3:**
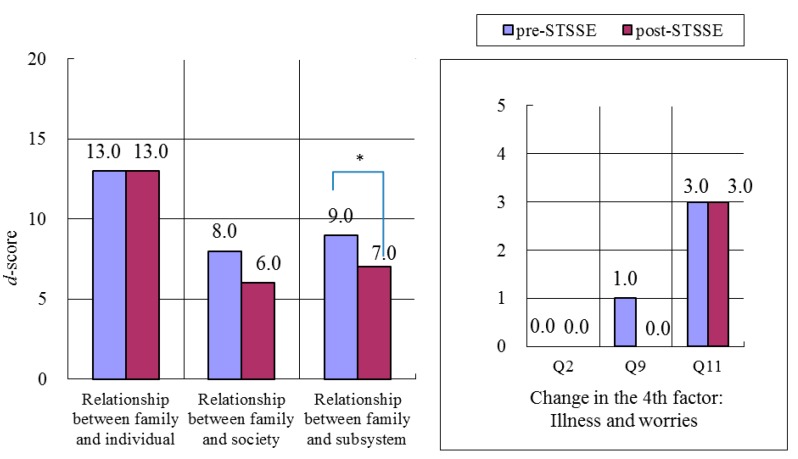
Changes in median FFFS scores for all 17 mothers before and after STSSE. Q2: Consultation and concern for relatives (excluding a partner); Q9: Using a medical institution and receiving health consultation; Q11: Worries about a child. *p* < 0.05.

**Figure 4:**
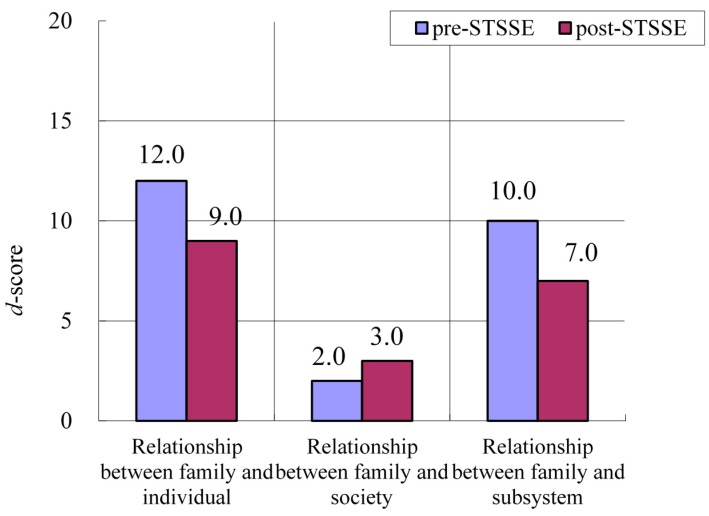
Changes in median FFFS scores for all nine fathers before and after STSSE.

Changes in FFFS scores are shown in Figure 3 and Figure 4.

#### 3.3.1. Mothers

Among the two factors making up “Relationship between family and subsystem (*p = 0.*019)”, significant improvement was seen in the *d*-score for “The 4th factor: Illness and worries” (*p* = 0.016). In addition, improvements were seen in the following sub-categories: “Q2: Consultation and concern for relatives (excluding a partner)” (*p* = 0.52); “Q9: Using a medical institution and receiving health consultation” (*p* = 0.10); and “Q11: Worries about a child” (*p* = 0.39) (Figure 3). In addition, “Relationship between family and subsystem” for mothers was improved after STSSE.

#### 3.3.2. Fathers

Improved FFFS scores for the following two items were seen for all nine fathers: “Relationship between family and individual” (*p* = 0.64) and “Relationship between family and subsystem” (*p* = 0.51); however, these differences were not significant (Figure 4).

### 3.4. Changes in CDQ Scores

#### 3.4.1. Mothers

The median CDQ scores increased for 11 of 18 items following the STSSE course in all 17 mothers; however, the increases were only significant for “Q12: Do you quarrel less with your family due to your child’s behavior?” (*p* = 0.01) (Table 4).

#### 3.4.2. Fathers

The median CDQ score increased for three of the 18 items after PTSS; however, no significant differences were observed (Table 4).

### 3.5. The Mothers’ Comments

In general, the comments of the mothers suggested the usefulness of STSSE. Most mothers reported that their child had more emotional control as a result of the joint SESSE activities, which required paying attention, expressing their emotions in words, and using suitable language when communicating with others (Table 5). Furthermore, because parents and children worked together using enhanced social skills, the validity of the STSSE course was demonstrated.

## 4. Discussion

### 4.1. Changes in Children

In this study, “Communication Skills” among children with ASD, which included “Hearing”, “Speaking”, “Assertion”, and “Talking”, showed significant improvement following STSSE. Some mothers of children who showed improvement made comments such as “My son has begun saying things to me like ‘this dinner is really good’,” and “My daughter was able to raise her hand and answer a question at school.” Since “Hearing”, “Speaking”, “Assertion”, and “Talking” can be used to evaluate the ability of an individual to draw conclusions, the improvement in these sub-items suggest that STSSE made it possible for children to use communication skills more effectively at school. Some parents reported that their children’s communication skills acquired through “Rehearsal”, “Modeling”, and “Homework” were also utilized at school. Teachers were informed of the content of the skill-training program together with a handout, and that their cooperation may also form the basis of the behavioral changes shown in the study results.

In contrast, no significant improvements were seen in the CBCL scores in this study. Although the direct aim of STSSE is the acquisition of social skills, a behavioral improvement was anticipated as well; however, based on CBCL scores, no behavioral improvement was seen in emotional control, coping, and peer relationships skills. Nonetheless, the improvement seen in communication skills may have resulted in reduced somatic complaints, suggesting that the children became able to express their mental state, including stress, anxiety, and conflict issues, more easily than before. In this study, some mothers of children who showed an improvement in expression of their mental state made comments such as, “My son does not bite his fingernails as much,” and “When I made a feelings sheet, the word “sad” began appearing from my child.” On the other hand, some mothers thought problems worsened; however, as shown in Table 5, some mothers of children who showed difficulty in relation to thought problems made comments such as “My child still seems to make decisions (either 0% or 100%, good or bad) based on emotions,” and “Even now he still always wants to be number one. And, his handwriting is sloppy because he wants to be the first to turn in his paper.” This result was thought to reflect increased recognition among parents of changes in their children’s thought processes that were previously difficult for them to understand. Overall, although an improvement in social skills was evident in the current program, no improvements anticipated by direct training to the children were seen in terms of externalizing problems. In addition, compared with PTSS, the effect of SST on the CBCL was insufficient. First, parents can more easily witness the direct effect of PTSS on their children’s behavior problems. However, in STSSE, which is a program that specializes in social skills, this effect is more difficult to see. Second, as demonstrated by the mothers’ comments, it is possible that there was a behavior change that could not be evaluated using the CBCL. Therefore, future studies should examine these issues further.

### 4.2. Changes in Parents

The *d*-score for “Relationship between family and subsystem” in the FFFS was improved after STSSE. This result suggests that strengthened support from a partner may be associated with increased life satisfaction and psychological well-being for mothers of children with ASD [20]. By virtue of STSSE training, mothers worried less about their children. Additionally, family functioning was improved through the increased opportunity for dialogue between parents.

In the STSSE course, mothers were recommended to share the materials and experiences obtained in the sessions with their partner, especially those regarding their children’s ASD-related characteristics. Most of the mothers reported that they practiced the same session with their partner at home and found time to talk about how to care for their children. Fewer children (cases 2, 13, and 16) who had parents with one of the negative parenting risk factors (critical behaviors or physical punishment) showed clinically significant improvements compared with those whose parents did not have a negative parenting risk factor [31]. It is clearly important for parents to build a good relationship with their partner in order to take good daily care of children with ASD.

As shown in Table 5, some mothers who noted that their family had started quarreling less frequently made comments such as “My daughter has come to understand the things that cannot be communicated with words and is more accepting of the things I say”, “We as parents no longer get angry as often”, “As parents, we have come to give her more steady praise”, and “As a parent, I have become more capable of listening to what my son has to say”. The median CDQ scores of fathers also increased in three of the 18 items after STSSE. These results suggested that, in addition to mothers of children with ASD to whom STSSE was directly applied, STSSE also had some favorable effects on fathers. This indirect effect might have been due to the fact that the program encouraged the mothers to share the content of the sessions with their children and partners at home as described above. Mclntyre (2008) [32] proposed that families with children with ASD tend to show more negative and inappropriate parent-child interactions than those with a child suffering from other developmental disabilities. He, therefore, emphasized the importance of training parents to focus on altering parent-child interactions. In our study, most of the mothers and children expressed unease when instructed to force their children’s social skills at the beginning of the STSSE course; however, the confidence in parenting increased as the STSSE sessions proceeded.

### 4.3. The Usefulness of STSSE

We developed and introduced an STSSE program based on the previously reported parent program, PTSS, primarily for children with ASD and their parents. STSSE differed from our original PTSS in the following aspects: STSSE, which was applied to child-parent pairs simultaneously, provides a chance for children and their parents to meet and talk together, feel sympathy for one another, and enhance their own self-esteem. In addition, STSSE places a greater focus on acquisition of social skills compared with PTSS.

This program did have some limitations. First, in this study, no improvement in externalizing behavior was observed in high-functioning children with ASD during the follow-up period; however, improvements were seen in social skills and peer relationships and, thus, externalizing problems may be alleviated in the long term. Alternatively, programs targeting younger children may be necessary, since earlier intervention for children with ASD has been associated with a better prognosis [33]. Second, some parents in this study reported difficulty interacting with other parents in their group, despite all parents expressing satisfaction after STSSE. Smaller groups or individualized training might be more appropriate for parents reporting these kinds of difficulties. Third, the sample size was too small to draw definite conclusions about behavioral and cognitive changes in children with ASD and their mothers, and the follow-up period was too short to detect long-term behavioral and cognitive changes. However, the consistent finding of increased confidence among caregivers in our PT-based programs, PTSS and STSSE, suggest their effectiveness.

## 5. Conclusions

This research evaluated the effectiveness of our newly developed STSSE by analyzing changes in mothers’ FFFS and CDQ scores and children’s SS-scale scores. Positive changes were seen in both parents and children; although these results are promising, an additional study with a larger sample size will be needed before they can be generalized to other populations.

## Figures and Tables

**Table 1:** Demographics of the families in this study.

Mothers (*n* = 17)		*n*	Mean (± SD)	Range
Age (years)			40.7 ± 3.3	35.0–49.0
Marital status				
Married		17		
Living with partner		17		
Educational level				
University education		11		
Work status				
Working part or full time		7		
**Fathers (*n* = 9)**				
Age (years)			43.7 ± 4.2	37.0–52.0
Educational level				
University education		5		
Work status				
Working full-time		9		
**Sibling**				
Sibling present		10		
Age (years)				1–29
Gender	Male	4		
	Female	8		
Sibling with developmental disorders		1		*
Sibling quarreling with child		5		
Sibling having trouble at school		9		

* Autism Spectrum disorder + Attention deficit hyperactivity disorder.

**Table 2:** Clinical characteristics of the children with autism spectrum disorder (ASD) in this study.

Case Number	Age (y/m)	Sex	Main Behavioral Problems	Test Battery	IQ/DQ	
					FIQ/Total DQ	VIQ/PIQ, P-M.C-A,L-S, *etc.*
1	7 y 4 m	M	2, 3, 4, 5, 6, 7, 8	Tanaka Binet	76	
2	8 y 3 m	F	1, 2, 5, 7, 9	KSPD	96	C-A100, L-S93
3	7 y 6 m	F	1, 2, 5, 7	KSPD	90	C-A92, L-S84
4	9 y 8 m	F	5, 7	WISC-III	60	VIQ63, PIQ64
5	8 y 3 m	F	1, 3, 4	WISC-III	92	VIQ96, PIQ89
6	9 y 10 m	M	2	WISC-III	83	VIQ76, PIQ57
7	7 y 6 m	M	2, 5, 7	WISC-III	101	VIQ104, PIQ99
8	9 y 6 m	F	2, 3, 4, 7	KSPD	68	C-A61, L-S76
9	9 y 8 m	M	1, 4, 5, 10	WISC-III	76	VIQ82, PIQ75
10	8 y 8 m	M	1, 2, 3, 4, 5, 7, 9	WISC-III	90	VIQ82, PIQ100
11	7 y 11 m	M	2, 3, 4, 5, 7, 9	WIPPSI	79	VIQ86, PIQ80
12	8 y 3 m	M	5, 7	KSPD		C-A86, L-S76
13	10 y 1 m	M	1, 2, 4, 7, 10	WISC-III	89	VIQ105, PIQ73
14	8 y 6 m	F	2, 5, 7	WISC-III	100	VIQ85, PIQ114
15	8 y 3 m	M	5	WISC-III	100	VIQ97, PIQ103
16	7 y 6 m	M	2, 3, 7, 9	KSPD	93	C-A88, L-S98
17	7 y 8 m	M	2, 3, 5, 6, 7, 9	KSPD	93	C-A86, L-S100

Main signs of childhood behavioral problems: 1. unease, 2. hyperactivity/impulsion, 3. emotional instability, 4. school problems, 5. the difficulty to follow directions, 6. abusive language and violence at school or in the home, 7. restless/attention deficit, 8. aggressiveness, 9. carelessness, 10. flashbacks. y: year; m: month. M: male; F: female. WISC-III: Wechsler Intelligence Scale for Children (third edition). KSPD: Kyoto Scale of Psychological Development; the KSPD was modified for children ranging in age from newborns to 14 years. Developmental age was estimated based on the psychologist’s observations and distinguishing features of the child’s behavior. WPPSI: Wechsler Preschool and Primary School Scale of Intelligence. Tanaka Binet:Tanaka–Binet Intelligence Scale V.

**Table 3:** Content and points of intervention for the 10 sesssions comprising an STSSE course for children with ASD and their parents.

Section	Children	Parents
Session 1: Understanding the greeting method	・Skill in addressing people (Keeping a suitable distance and maintaining eye contact, turn-taking in speaking)	・Understanding the general features of ASD and the principles of social skill training ・Identifying good social behavior in the child and understanding ways to give praise ・Understanding the skill of initiating engagement and cooperating with friends at school, *etc.*
Session 2: Understanding how to extend play invitations	・Skill at the time of starting a peer relationship (Adjusting speaking volume, suitable speech, and proper methods of refusal)	・Learning how to encourage the behaviors needed by children and the required skills toward appropriate interactions with friends and relations
Session 3: Understanding how to ask for help	・Skill in asking others for assistance when necessary ・Conveying gratitude with respect to speaking volume, suitable speech, and proper methods of refusal	・Learning how to encourage the skill required to ask for assistance
Session 4: Understanding warm behavior	・Affirmative influence ・Classification of “affable” and “cruel” words	・Noticing and monitoring appropriate behaviors and rewards ・Rewarding the child for their appropriate behaviors through praise, attention, and physical contact
Session 5: Understanding warm behavior	・Affirmative influence ・Learning scene-specific “heart-warming words”	・Understanding the skills required for conversation with a good friend or relation
Session 6: Understanding conversation skills	・Skills regarding turn-taking and eye contact in speaking	・Utilizing self-control techniques for emotional management ・Understanding how to urge the child to use the skills aimed at acquiring self-control
Session 7: Understanding emotions	・Matching expressions and feelings by learning about the relationship between an event and its associated emotions		
Session 8: Understanding emotions	・Levels of feeling ・Learning about grades of feeling	
Session 9: Understanding emotions	・Feelings of control ・Relaxation techniques	
Session 10: Farewell party	・Participating in a game with a high level of difficulty ・Affirmative evaluation of participation	・Reviewing all previous sessions and preparing an environment designed to reduce inappropriate behaviors ・Learning how to cooperate with school and society

**Table 4:** Total scores and subscale socres for mothers and fathers on the confidence degree questionnaire (CDQ) for families.

CDQ Questions		Mother (*n* = 17)					Father (*n* = 9)				
		Pre–STSSE		Post–STSSE		*p*	Pre–STSSE		Post–STSSE		*p*
		Median	IOR	Median	IOR		Median	IOR	Median	IOR	
Q1	Do you watch your child’s growth without becoming impatient?	3	3–4	4	3–4	0.83	4	4–4	4	3.5–4	0.41
Q2	Do you accept your child’s diagnosis of ASD?	4	4–5	4	3.5–5	0.68	4	3.5–4.5	4	3.5–5	0.49
Q3	Do you let your child do what he/she can do by him/herself?	4	4–5	4	4–5	1	4	4–5	4	3.5–4.5	0.16
Q4	Do you praise your child once or more a day?	4	4–5	4	4–5	0.74	3	3–4.5	4	2.5–5	0.41
Q5	Do you prepare a place where your child can relax?	4	4–5	4	4–5	0.41	3.5	3–4	4	3–5	0.32
Q6	Do you help your child to make friends?	4	3–4.5	4	3–4	0.53	3	2–4	3	2–4	1
Q7	Can you cope with your child’s inappropriate behavior?	4	3–4	4	3–4.5	0.09	4	2.5–4	3	2.5–4	1
Q8	Do you communicate adequately with the school about your child’s problems in school?	4	3.5–4	4	4–5	0.07	4	2.5–4	3	2.5–4	0.68
Q9	Do you blame yourself less for having a child with ASD?	4	3–4.5	4	3–4.5	1	4	3–4	3	2.5–4	0.24
Q10	Are you less worried about your child?	3	2–4	3	2–4	0.43	3	3–4	3	2–4	0.32
Q11	Do you spend time on your own health or enjoyment?	4	2.5–4.5	4	2.5–4.5	0.61	3	3–4	3	3–4	1
Q12	Do you quarrel less with your family due to your child’s behavior?	3	2.5–4	4	3–4	0.01 *	3	2–4	4	3–4	0.53
Q13	Do you ask your family members to assist your child?	3	2–4	3	2–4	0.59	3	2.5–3.5	3	2–4	0.79
Q14	Do you consult your family or friends about your troubles and not worry by yourself?	4	3–5	4	3–5	0.41	4	3–4	3	3–4	0.23
Q15	Do you share your feelings with families who have children with a similar problem?	4	4–5	4	4–5	0.18	3	3–4	3	2.5–4	0.26
Q16	Do you utilize medical facilities, and school and consultative organizations if required?	5	4–5	5	4–5	0.71	4	3.5–5	4	3–4	0.26
Q17	Do you understand your child’s behavior and ideas/feelings/thoughts?	3	3–4	4	3–4	0.32	3	2.5–4	3	3–4	0.48
Q18	Do you feel happy being with your child?	4	4–5	5	3.5–5	1	5	4–5	4	3.5–4.5	0.06

*: Significant *p* < 0.05.; Interquartile range: IQR; CDQ questions: How much confidence do you have in the following matters? Please circle the number most applicable to your present feeling on a scale from 1 to 5, as indicated below. 1: I am not confident, 2: I am slightly confident, 3: Neutral, 4: I am somewhat confident, 5: I am confident.

**Table 5:** Comments from mothers at the end of each session.

Subject Number	Mothers’ Comments
1	When I made a feelings sheet, the word “sad” began appearing from my child. It was a good lesson for me as well, and I would like to continue.
2	Since attending STSSE, my child has been increasingly inviting friends to the house for short visits. I am glad to see that.
3	My daughter has come to understand the things that cannot be communicated with words only and is more accepting of the things I say. Her sister has reported to me that she was doing her homework without being told.
4	My daughter has come to be able to control her emotions. She has become able to process things well after her emotions explode. I am enjoying it as well.
5	I did not know what my daughter was thinking before, so it was good to understand her thoughts in this exercise. I realized that my daughter was also paying attention to me. There are limits to what parents can communicate to children. Neither my spouse nor I have friends, so that’s an issue that needs to be addressed.
6	My son has become better at how he deals with things after he becomes angry.
7	It was enjoyable for my son and I. My son now has fewer quarrels with his friends.
8	My daughter was able to raise her hand and answer at school. Although, it was only two times, it still made me happy. I want to continue practicing so that I can communicate my feelings with words using the basic pattern we learned at STSSE.
9	Even when trying to ask my son why he thought something, he had built an emotional wall and I could not get in. My son seems to have made a decision to not talk about what happens at school, and will not tell me. I received a call that there might be trouble with the sixth grade students at the school sports festival; my son always wanted to be in the first position. But on the day of the festival, everything was fine. In the running race, even though he was third, he lined up in the third place position smoothly without being told to do so.
10	I really felt that STSSE was effective for us as parents. I was even asked at my son’s school if I was doing something different. My son does not bite his fingernails as much. A major issue now is emotional control, and we are looking for solutions as a family. We parents learned something too.
11	I understood that my son needs to grow with praise. I also understood that in an organized environment, he can make friends. He still seems to make decisions with emotions either 0% or 100%, good or bad.
12	My son has come to understand troubled looks on people’s faces. I hope he will become able to express his emotions in words. We as parents no longer get angry as much.
13	With his disorder, my son does not understand the difference between himself and others; and I thought that by making a measure (scale) for each emotion and showing him visually, it would be easier for him to understand and to do self-analysis.
14	When I ask about school or other things that my daughter does not want to talk about, she will tell me and resolve her feelings a little if we move to another place to talk. I have learned the keys to using STSSE. As parents, we have come to give her more steady praise.
15	My son does not seem to be good at saying things that make others feel good, but he has begun saying things to me such as “this dinner is really good.”
16	My son said that the STSSE games were fun. Even now he still always wants to be number one. His handwriting is sloppy though because he wants to be the first to turn in his paper; this is something we are working on now.
17	My son has become a master of the multiplication table and seems to have confidence in himself. Not being understood by others causes stress in him, and I want him to be able to say things. I wish he were able to switch moods when something is bothering him. As a parent, I have become able to listen more to what my son says, and have become able to be a little more reassured.

STSSE: simultaneous training for children with ASD and their parents with a focus on social skills enhancement.
